# Editorial Department

**Published:** 1884-10

**Authors:** 


### The Bistoury.

ELMIRA, N. Y., OCTOBER, 1884.

The BISTOURY is published Quarterly, upon the first of April, July, October and January, at Fifty Cents a year in advance.

### Editorial Department.

CHOLERA COCCUS.—The French medical journals assert that a microscopical examination of the water supply of Marseilles, France, shows it to be thoroughly stocked with the special coccus of cholera.

NOT RESPONSIBLE.—We repeat a suggestion we made in the July number of the BISTOURY, that we may sometimes differ with our regular contributors in their ideas on social, political or medical subjects, but we always estimate views from such sources as worthy of consideration.

AT WORK AGAIN.—The "old doctor," who for months, has been very seriously ill—from inflammation of the brain—is happy to announce that he has fully and entirely recovered. This announcement is made in answer to kind letters of enquiring friends, sparing us a deal of letter writing.

A NEW SAW.—Said an estimable Quaker lady to us, recently, while discussing the numerous and sickening scandals that are applied to the Nation's presidential candidates: "If all our sins were written upon our foreheads, does thee know it would be a very great novelty to see a man wearing his hat upon the back of his head?"

PHARMACO-DYNAMICS.—A century ago, the pharmaco-dynamic properties of drugs were generally known only from some accidental cases of poisoning, or from the effects produced by an excessive dose of the drug. One drug was simply known as an emetic—more or less mild or severe in its operations, but still only as an emetic; another drug as a febrifuge; another as a sudorific; another as a diuretic; another, again, as a rubefacient; another as an antiphlogistic—generalizations that led to, and confirmed the speculative practice of former times. Modern therapeutics is an attempt to dissipate the old, arbitrary groupings of drugs, and to give to each one an individual action.

THE NORTH POLE.—We cannot, especially at this season, with a market overflowing with good things, entertain for an instant the horrible ideas, suggested by a reference to canabalism, but we understand plainly the necessity under some and almost all circumstances for the commander of an expedition out of reach of all law and civilization to protect the lives of those under his charge by any sacrifice necessary, even should it be life itself. Our sympathies go out to all driven to such extremeties as those of the Greely expedition party, not only in their physical sufferings and horrors, but especially in the moral gauntlet they have been forced to run since their return.

Why parade such horrors before the world? "Necessity knows no law," and there are many things which are done under that rule that are better not referred to.

FATHER STACK.—It will be remembered that a few years ago this gentleman, then a Catholic Priest of considerable eminence, presiding over a church in our neighboring city of Williamsport, had a legal controversy with his Bishop, in which the Priest showed a disposition to maintain his rights as a private citizen, said rights having been ignored by his Bishop. The conflict waxed long and strong, in which the Priest eventually gained from the courts that which he was deprived of by the Bishop. Well, Father Stack made a pilgrimage to Rome, asked pardon of the Holy Father for suing his Bishop, was forgiven and is now fully installed in a church of his faith, in the far West. It is said however, that his little tilt with the Bishop was the means of modifying the power heretofore exerted by the officers of the church over the priesthood in the United States.

SAXON.—We glory in the good old saxon race and its uncompromising stern old language, but men and times change, so that we feel, to speak mildly, slightly shaken when we glance over the morning or evening newspaper and find somebody who is suspected of mendacity, called so in plain, unvarnished saxon, or somebody else who happens to have the misfortune of being a candidate for some office, accused of having a family without the intervention of church or civil authority, in terms which are so closely allied to the old saxon that we put away the paper before the ladies of the family are scandalized by it.

It is to be hoped that before we enter into another presidential campaign, the editorial corps will get together and establish some code governing these matters which will at least protect their readers from such improprieties of language.

---

LET US MIGRATE.—It would be well for those who have a tender regard for the sensibilities of their wives and daughters, and who can afford the costly expenditure of means, to leave our country with them immediately after the Presidential nominations are made for another canvass. The nastiness of the present campaign is past endurance. We might be spared some of the disgrace, consequent upon the filthy and criminal charges against our presidential candidates, if both parties were to acknowledge, at the time of the nominations, that the nominees offered to the people are seducers, robbers, liars and murderers—entitled also to other selected criminal titles—but then and there agree not to discuss, in the newspapers, the particulars of their offences against decency.

As the matter now stands, we are not only suffering the humiliation of living in a country that will countenance such men, as the nominees are represented to be, but are subjecting our families to the evil influences of a scandalous and disgusting literature.

---

THE VALUE OF THE MICROSCOPE TO THE PHYSICIAN.—Every year, the microscope becomes of more value to the physician in determining the nature and cause of disease, until now, it is an absolute necessity in the thorough equipment of the medical practicioner of medicine and surgery. In determining the character of tumors and cancers, of secretions and discharges on and from the body. In examining the blood—by means of which various diseases have been detected, and the proper method of medication determined upon. As, for instance, in prescribing for rheumatism. In the blood of rheumatic individuals has been seen, under the microscope, oxalate of lime, phosphate of lime soda crystals, uric, hippuric, and other acids, masses of fibrin, etc. The corpuscles themselves have been seen distorted and contracted and the number of white corpuscles determined and certain symptoms due thereto accounted for. It is plain to be seen, how valuable such information must be to the physician in determining the line of treatment to be adopted in restoring his patient to health.

---

PURULENT INFLAMMATION OF INFANT'S EYES.—Our attention has been directed, several times recently, to the carelessness of parents, in giving proper and early attention to a certain inflammation of the eyes of the new born infant. A report of the proceedings of "The Ophthalmological Society of the United Kingdom," shows that the society sent printed instructions to nurses and midwives, in charge of infants, as follows: "If the cheeks and eye-lids become red and swollen, or begin to run with matter, within a few days after birth, it is to be taken without a day's delay to the doctor. The disease *is very dangerous*, and, if not at once treated, may destroy the sight of both eyes."

When it is known that the reports of the blind asylums show that nearly forty per cent. of the cases of incurable blindness are the direct result of *ophthalmia neonatorum*—the disease just described—our mothers should be informed of its danger that they may make early application to the physician for their infant's relief.

---

THE NEW MANIA.—When the impulsive American becomes vaccinated with the virus of any craze, the innoculation is so thorough and the resulting fever so pronounced, as to exclude all other ailments. In fact, he gives his whole mind and all the energy of his physiology to it. Witness the marvelous fascination that the roller skating rinks have exerted over the young and old people, alike. All classes and conditions of men and women yeild to the captivating influences of the exhilerating sport. And why should they not ? The exercise is healthful, the motion graceful, the companionship proper, the hours unobjectionable, and the influences and surroundings as uncontaminating to the moral culture of our young folks as any large gathering of respectable people can be. The arguments that have been advanced, particularly by the clergy, to prove certain immoral influences that are supposed to pervade these assemblies, do not seem to us to be logical. If it be true that since the opening of the rinks, the numerous drinking saloons of our city have been almost wholly deserted, and that the usual attendants upon these places have migrated to the new resorts for amusement, surely, this circumstance should be placed to the credit rather than to the debit of the rinks. Neither is it fair to blame the rinks with affording the former saloon visitors an opportunity to mingle and associate with our wives and daughters, when precisely the same conditions are gladly sought for in the churches and prayer-meetings. The exact truth seems to be that, under proper restrictions and the supervision of parents, the skating rinks are admirable and suitable places for our children to congregate, affording agreeable companionship and healthful exercise, accompanied with as little danger to morals as in any large assembly of miscellaneous people, and as exempt from accidents to life and limb as any exercise of equal value.

---

BANKS.—We have perused with much satisfaction an extract from a paper read at Saratoga before the American Bankers Association. The subject taken was "The Conditions of Safe Banking," the author, Mr. B. B. Comegys, of Philadelphia. There are so many points in the extract which will interest and be suggestive to the readers of the BISTOURY that we give it entire :

"A bank may be said to be in good condition when it has an adequate capital (not too large); a contingent fund at least half as large (and no suspended debt or overdue paper); when its deposits are free of interest and three or four times the amount of its capital ; when its dealers supply it with business paper to the extent of its needs; when liberal salaries are paid to its officers and clerks ; when there is a trained man in reserve for every position that may become vacant; when there is a pension fund adequate to the comfortable support of its worn-out clerks; when it has a Board of Directors who are not content to be mere figure heads, but who understand their business and remember their qualification oaths—directors who count the cash frequently, and without notice to anybody; who insist that every person employed in the bank shall take a vacation of at least two weeks every year, at which time another person shall do his work; and who believe in this dogma, that 'nothing is good enough that can be made better.'"

Without referring to the monetary portions which more properly belong to the columns of a banker's magazine, we would commend two points ; first—that a trained man should be in reserve for every position which may become vacant. Second :—that every person employed in the bank shall take a vacation of at least two weeks every year, at which time another person shall do his work.

It would be a work of supererogation to point out the advantages of these rules to a bank ; but perhaps some may fail to apply one at least of them to general business as should be done.

Apart from the recuperation to mental and physical faculties to be gained by the clerk and its reflex action upon the master's interests, the necessary investigation of the affairs of the desk and the methods in use there, by a second party, must ensure either an honest administration or the speedy discovery of fraud.

How many merchants with this hint, can look back upon the losses sustained from dishonest clerks in whom at one time they placed implicit confidence, and whose deep laid schemes might have been thwarted by an enforced vacation? Perhaps Mr. Comegy's paper may save both masters and clerks in the future.

---

MUTUAL AUTOPSY.—We have been invited to join a society in New York, organized for mutual autopsy. There is an element of grotesqueness in the suggestion that led us to suspect that Mark Twain would be found at the head of the organization. After reflection, however, the absurdity of the proposition disappears and the wisdom of the move becomes apparent. Each member endows, in writing, the society with his body, to be dissected, examined and experimented with, after his death, by the surviving members. The society's apartments are said to be elegantly furnished, where daily and nightly meetings are held, and where the jolly fellows congregate to canvas the question as to who is to be the next subject for the dissecting table. Any fellow exhibiting a cough, a pain in the liver, or symptoms of "Bright's disease," is wined, dined, and cigared by all the others ; every possible opportunity taken advantage of to hasten the termination of his ailment, while they quiz him as to his symptoms, manifesting the greatest delight and satisfaction when he is no longer able to leave his bed to join the meetings of the club. Then, they send delegations to visit him daily, making minute inquiries as to his symptoms and exhibiting the new instruments to be used in carving him up. They show the staining fluid to be injected into his arteries and veins, and acquaint him with the particulars of the beautiful sections and mounts they mean to make of his double-injected kidney, liver and lungs. They explain how they mean to harden his brain in chromic acid, wash it in alcohol, and stain it in picro-carmine. They show the handsome microtome, imported from Vienna, with which they mean to slice his brain, and illustrate its smooth working and its clean cutting upon a chunk of the family cat's brain. This exhibition, of course, delights the sick man, gives him the most pleasant of reflections, quiets his nervous system, and induces him to quit his body as speedily as possible that his dear friends may the more quickly carve, slice and shave his several organs, while with tear-blind eyes they discuss his whims, peculiarities and loveable traits of character that so endeared him to them.

Yes, it is a great institution, is that mutual autopsy society. It spares all expense of a funeral; affords an assurance that your body will not be stolen and dissected by strangers, but that it will be loveingly handled by admiring friends. Cremation societies are likely to receive a great rebuff from the advent of this new and cheerful society for disposal of the dead. We wish it the success it deserves; but, as our brain, liver, stomach, kidneys, etc., are in especially fine working order at this writing, we assure these patient and considerate investigators of anatomical, physiological, and pathological science, that nothing new is to be gleaned from prying into our private affairs, and as the partner of our joys and sorrows once assured us that she had as soon "hug a bag of clothes-pins" as ourself, it will be seen that we are better calculated for a skeleton than a magnificent specimen in anatomy. We sorrowfully decline, with many thanks.

---

SANITATION.—Recently, the board of health of this city, gave orders for the removal of certain hide-storing establishments, located on one of the principal thoroughfares. These conservatories of perfumes permitted the escape of much of their fragrance into the surrounding air, much to the annoyance and great disgust of the passers-by, who became clamorous for their transfer to less populous precincts. The proprietors of these cutaneous repositories dissented from the opinions promulgated by our health guardians and stoutly maintained that their peculiar goods were not detrimental to health, offering in illustration and confirmation of this belief, an inspection of their own personal physiologies, in which they severally exhibited a rotundity of person and a weight of corporosity, certainly not equalled by any member of the board of inspection. This argument, although seemingly quite convincing, failed to impress the board favorably, the members thereof insisting upon an observance of their order and a removal of the goods—noxious vapors and all—within the prescribed limit of thirty days.

In this demand, the board of health is right, since the noxious gases and vapors arising from decomposing animal tissues, and mingling with the air we breathe, cannot be otherwise than detrimental to public health. This has been abundantly demonstrated by sanitary writers again and again, so that reading people should be tolerably conversant with the arguments illustrative of the scientific facts involved. The microscopic fungi, known to be provocative of certain diseases, are found in great abundance, distributed upon the window-glass, over the floors and upon the furniture of the rooms where these stinking hides are kept. They soon dry, when they are blown about, hither and thither until some moist lodging place—like the throat and lungs of the passers-by—is found, when they immediately assume life and activity, to poison the individual who becomes their host.

Recently, we had occasion to examine the water and ice used in a family in this city, where three cases of typhoid fever existed. While the well water was far from being potable, the ice was filled with myriads of pupæ of a very small insect. When the ice was melted, these creatures at once became lively, collected upon the surface of the water, in what seemed to be a white scum, and were seen, under the microscope, to hop about in the liveliest manner. They had mandibles, three pairs of legs, armed with claws, two black eyes, and eight rings to an elongated body. They resembled the familiar "grub" found in decaying wood.

We carefully skimmed these from the water, placed them in a small quantity of milk and fed them to a perfectly healthy kitten. The kitten at once fell ill, became languid and puny, vomited, had bloody discharges from the bowels, filled with mucus shreds. The discharges contained the identical creatures fed from the ice. In fourteen days, the kitten died. We were prevented, at the time, from making a post-mortem examination of our poor little martyr to science, but doubt not we would have found the intestinal canal lined with the pupæ and their eggs—the direct cause of kitty's sickness and death.

While these creatures are not the same as found among the decomposing hides and floating in the air of the hide-storing establishments, they show, in a very emphatic manner, the danger that lurks in microscopic life, when permitted to enter the body. We cannot be too careful in selecting the water we drink and in keeping the air we breathe, pure and stenchless.

POISONOUS STOCKINGS.—Beware of wearing woolen stockings dyed with brilliant, aniline colors, especially scarlet. These colors are highly irritating to the skin, in many cases, producing serious consequences. A gunning friend returned from the field having worn red stockings inside of rubber boots. His feet perspired profusely, his skin partook of the same stain as that of the stockings, and he is now laid up with a very sore foot and leg—too sore, indeed, to adequately compensate for the fourteen grouse that rewarded his day's shooting.

We have seen several cases of similar poisoning, in children whose feet and legs have been ornamented with these highly colored stockings. Beware of them.

---

A NEW PROPERTY OF THE RED BLOOD-CORPUSCLES.—At a meeting of the Italian Medical Association, Dr. Fano related his experiments with peptone, and spoke of the rapid cessation of the reaction of peptone in the blood. He demonstrated the transformations of peptone absorbed by the digestive tract or transfused into the blood-current, and how peptone may be transformed and stored up by the morphological elements of the blood. The transformation consists in a process of dehydration, by which the peptones are changed into coagulable albuminoids. The active elements of this transformation are the red corpuscles which, assimilating the peptones that enter into the circulation, increase the specific weight. It is probably to the potash salts which the red corpuscles contain that this dehydration of the peptones is due, by which they are transformed into globulin. For this process to take place, the presence of oxyhæmaglobin is an indispensable condition. The stored-up albuminoids serve as a reserve supply of aliment, which is given up to the tissues as required.

---

ENDOWMENT OF A PROFESSORSHIP.—There is being made an effort to raise a sufficient sum of money, by subscriptions from the medical fraternity, to endow a "Gross professorship of pathological anatomy," in Jefferson Medical College, of Philadelphia.

This is all well enough, in fact, a commendable enterprise, but would not the profession subscribe more willingly and abundantly should Prof. Gross's heirs take a monied interest in said endowment? Prof. Gross was a very famous, skillful and honored surgeon, known the entire world over. By reason of these qualifications, he became deservedly very wealthy, leaving an estate which we have heard estimated not less than half a million dollars. Certainly, his well-to-do family might spare from this large sum, ten or twenty thousand dollars to endow a professorship bearing the honored name of the head of the family. One of small means might subscribe the more generously and willingly to see a wealthy family, more interested in such an honor than any other parties possibly can be, head the list of subscription, with a generous sum, from their very large means.

---

AN ARGUMENT FOR EVOLUTION.—Did you ever witness the manœvers of a hen in seeking to gain her roosting place upon some favorite tree? She first seeks a slight elevation upon the fence, this she paces backward and forward, casting her eyes aloft, diligently inspecting the coveted limb and occasionlly making a false motion as though about to fly, but thinking better of it, continues her pacings, squattings and tumblings from the fence, until an entire hour is wasted in finally getting to roost. We have seen gentlemen, entitled to know, declare that these demonstrations of the hen in preparing for bed are aptly imitated by the ladies—their wives. "There!" exclaimed a gentleman, in our hearing, while watching the tedious performance of a chicken in securing a perch for the night, "there, that is just the sort of work my wife makes of it. She will walk clear across the room to lay her hair-pins there; the same distance to deposit her switch; to another point to hang her skirts; to the bureau to carefully roll her corset; to the wash stand to paddle in the water; then, seeking a particular chair—passing at least a half dozen equally as comfortable, will slowly and deliberately remove her stockings; and, after you have awakened from your first nap, you will find her still there, contentedly rubbing her toes, or scratching her head." Can it be that our women were evolved from the hen? We have seen very pretty, graceful and handsome plumaged birds that might lead us so to suppose, while this bed-seeking exhibition might be confirmatory of the theory. Then, if it be true that man sprang from the monkey, certainly the ladies can take no exception to being classed with the birds.

